# Greater toxic online disinhibition and lower consistent online self-presentation contribute to the perpetration of cyber dating abuse

**DOI:** 10.3389/fpsyt.2025.1716617

**Published:** 2026-01-12

**Authors:** Rebecca Ritchie, Maria Limniou, Sarah Gordts

**Affiliations:** Department of Psychology, Institute of Population Health, University of Liverpool, Liverpool, United Kingdom

**Keywords:** cyber dating abuse, online disinhibition, online self-presentation, digital dating abuse, technological intimate partner violence

## Abstract

**Background:**

While online interaction can support romantic relationships, it may also increase the risk of Cyber Dating Abuse (CDA - abuse perpetrated via technology between romantic partners). CDA consists of two types of aggressive behaviours: Direct Aggression and Cyber Control and Monitoring. Past research explores overall CDA perpetration with relationship satisfaction. However, there is limited evidence for the association between computer-mediated communication variables (i.e., Online Disinhibition, Online Self-Presentation strategies, and online platform use) and the two variations of CDA.

**Methods:**

A cross-sectional online questionnaire study was conducted using opportunity and snowball sampling. Overall, 326 participants aged 18 to 68 years old were recruited (*M* = 24.47, ± SD = 8.52; 71.50% women). Participants were required to be over 18 years old, have at least one previous romantic relationship, and be English-speaking social media users.

**Results:**

Weighted Least Squares multiple regressions indicated that “toxic” Online Disinhibition and “ideal” Online Self-Presentation positively predicted Direct Aggression and Cyber Control and Monitoring behaviours. “Consistent” Online Self-Presentation was negatively associated with Cyber Control and Monitoring only. In relation to Direct Aggression, “benign” Online Disinhibition was a negative predictor, whereas general online platform use frequency was positively associated. Relationship satisfaction was not significantly associated with CDA.

**Conclusion:**

Computer-mediated communication variables may exert greater influence on CDA perpetration than relational variables, as the anonymity and invisibility afforded by the online environment contribute to CDA. The findings highlight the importance of raising awareness regarding how the online environment can facilitate abusive behaviours.

## Introduction

Online communication is increasingly used to initiate and maintain romantic relationships (1). However, this digital intimacy can facilitate abusive behaviour (2). Cyber Dating Abuse (CDA) refers to abuse perpetrated via technology between romantic, dating, or ex-partners (3). CDA consists of Direct Aggression, whereby technology is used to intentionally harm a partner, and Cyber Control and Monitoring, where it is used to control or surveil a partner (4). It is associated with outcomes comparable to offline abuse, including low self-esteem, depression, and suicidal thoughts (5–7). Despite its detrimental effects, CDA is sometimes normalised, perceived as a sign of care or concern (8), with prevalence estimates suggesting that nearly half of young adults have experienced it globally (9). A recent meta-analysis by Li et al. (10) found that 43.4% of adults have been victimised by CDA and 44.6% have perpetrated it, which highlights the scope of the issue.

Although CDA is enacted via digital platforms, existing research has predominately emphasised relational and demographic predictors, leaving the role of computer-mediated communication (CMC) factors relatively underexplored. To address this gap, the present study investigates CDA perpetration through the lens of key CMC variables, specifically Online Disinhibition (OD), Online Self-Presentation (OSP), and patterns of online platform use, given their established associations with online aggression more broadly (i.e., 11–13).

Online Disinhibition Theory proposes that individuals often exhibit less restraint in digital environments than they would offline (12, 14, 15). This manifests in two distinct forms: “benign” OD characterised by increased openness and altruism and “toxic” OD, marked by hostile, critical, or aggressive behaviour (12). Features of online communication such as anonymity, invisibility, physical distance, and asynchronous interaction, contribute to disinhibition by weakening the psychological link between online actions and offline identity (12). These conditions can diminish empathy (16) and increase the likelihood of online aggression (17), consistent with OD theory.

Empirical evidence suggest that anonymity and “toxic” OD are associated with various forms of online hostility, including cyberhate and cyberbullying (18–21). Within the context of CDA, qualitative studies suggest that the perceived invisibility and physical separation inherent in digital platforms embolden perpetrators (22). Duerksen and Woodin (23) found that OD was positively associated with CDA, even after controlling for offline Intimate Partner Abuse (IPA), highlighting OD’s independent contribution to relational aggression. Given the robust correlation between IPA and CDA (24–27), OD may be a critical CMC factor in understanding CDA perpetration.

Additionally, Sánchez-Hernández et al. (28) explored the associations between OD and the distinct forms of CDA. This is important given that the outcomes of both forms of CDA differ: Direct Aggression is performed to explicitly harm a partner, whereas Cyber Control and Monitoring is performed to control and surveil a partner (4). OD may therefore affect the various forms of CDA with different strengths. Sánchez-Hernández et al. (28) found that OD positively predicted Direct Aggression only, whereas a former study by Maftei and Dănilă (29) reported that both greater “toxic” and “benign” OD were significantly correlated with higher CDA perpetration. These findings indicate a more complex relationship between disinhibition styles and CDA behaviours. Taken together, OD is emerging as a causal factor in CDA perpetration; however, less is known about the relationships between “benign” OD and “toxic” OD, and the distinct forms of CDA (i.e., Direct Aggression and Cyber Control and Monitoring). Both forms of disinhibition differ in their characteristics, with “toxic” OD being related to online aggression and “benign” OD being linked to online prosocial behaviours (12). Thus, “toxic” OD could be vital in the occurrence of both forms of CDA while “benign” OD may buffer against it.

A closely related yet distinct construct in the digital behaviour landscape is Online Self-Presentation (OSP), the strategic curation of one’s digital persona to shape others’ perceptions (30). Fullwood et al. (31) discussed three primary OSP modalities: the “consistent” self (alignment between online and offline identities), the “ideal” self (aspirational traits projected online), and the “multiple” selves (context-dependent personas across platforms). The malleability of OSP is amplified by the online environment, which enables selective concealment and identity experimentation. This allows individuals to express traits or behaviours that diverge from their offline persona, often aligning with personal or social goals (11, 32, 33).Individuals often enhance their OSP to elicit positive evaluations (15, 32), but discrepancies between these selves (i.e., “ideal” and “multiple” selves) may also contribute to maladaptive behaviours online, including CDA. For example, an inauthentic or inconsistent OSP, where online portrayals deviate significantly from offline realities, has been empirically linked to various forms of online abuse, including cybertrolling (11), intimidating behaviour (34), and derogatory comments (35, 36).

These behavioural outcomes can be partially understood through the lens of OD. Enhanced anonymity and reduced accountability may lower social evaluation risks, thereby emboldening individuals to act aggressively or impulsively (33). Moreover, the ongoing monitoring required to maintain inconsistent OSPs, especially when juggling “ideal” or “multiple” selves, may impair behavioural self-regulation (37), further increasing susceptibility of CDA. Conversely, Hu et al. (15) argue that online aggression may not always stem from disinhibited or inconsistent personas. Instead, such behaviours might reflect someone’s true/”consistent” self, a more authentic expression of underlying traits that are suppressed offline due to social constraints. This challenges the assumption that CDA is always a product of identity distortion, highlighting the need to measure both consistent and inconsistent OSPs. In sum, the interplay between OSP, OD, and CDA underscores the importance of examining how digital identity management strategies, especially those marked by inconsistency, can shape online behavioural outcomes.

Finally, the frequency and the purpose of online platform use may play a significant role in the perpetration of CDA. As internet use becomes increasingly embedded in daily life, individuals face mounting pressure to disclose personal information online (32). Social media platforms in particular offer unprecedented access to others’ lives, reshaping norms around intimacy and privacy. This heightened visibility, especially of romantic partners (38), can intensify relational scrutiny and surveillance, particularly in contexts where oversharing is prevalent (32). Such exposure can amplify perceived relationship threats, including seemingly innocuous actions, such as a partner liking another’s post or appearing in photos with other potential romantic interests, which can evoke jealously and prompt controlling behaviours (39, 40). The lack of contextual cues in online communication (e.g., tone, intent) further risks misinterpretation (41–43), increasing relationship anxiety and triggering controlling behaviour (38, 44). This dynamic may escalate into a vicious cycle of electronic surveillance where jealousy and suspicion reinforce abusive monitoring practices (38, 43–47), a pattern increasingly recognised as a form of CDA.

Therefore, online platforms do not merely enable these behaviours, they may actively facilitate CDA (48). Frequent smartphone and dating app use have been linked empirically to both Direct Aggression (i.e., online sexual harassment, posting sexual content of a partner) and Cyber Control and Monitoring (i.e., surveillance of a romantic partner) (26, 49, 50). These behaviours reflect a broader trend in which digital environments lower behavioural inhibitions and provide tools for covert or persistent aggression. Despite these associations, few studies have differentiated the impact of online platform use purposes, such as general social networking (e.g., connecting with friends, professional networking) (51) versus dating-related purposes (e.g., meeting new partners, maintaining romantic relationships) (52). This distinction is critical, as general-purpose users may engage with online platforms more frequently, increasing exposure to relational triggers and the opportunity for CDA. In contrast, dating-purpose users may use online platforms less often but in more emotionally charged contexts, potentially heightening vulnerability to jealousy and control dynamics. To address this gap, the current study examines the frequency of online platform use by purpose, rather than by platform type, to better understand how different usage patterns may contribute to CDA perpetration. This approach allows for a more nuanced analysis of how digital behaviours intersect with relational aggression in online settings.

Building on the preceding discussion, OD, OSP, and frequency and purpose of online platform use may be critical CMC variables in understanding CDA perpetration. Each factor contributes uniquely to the digital conditions that foster online aggression, whether through identity experimentation, behavioural deregulation, or relational surveillance (11, 16, 18, 20, 21, 26, 34, 49, 50). To further conceptualise the role of these CMC variables in relation to CDA perpetration, this study incorporates relationship satisfaction, a well-established predictor of CDA. Lower satisfaction within romantic relationships has consistently been linked to increased CDA perpetration (26, 53–55), often by intensifying negative emotional states, such as anger, distress, and resentment (56–58). These emotions are frequently directed toward the romantic partner and may be exacerbated by the ambiguity and hypervisibility of online interactions. For instance, dissatisfaction may provoke heightened anger (56, 59), which in turn elevates the risk of CDA perpetration (44, 54, 60, 61).

Despite growing research interest in CDA, the role of CMC remains largely unexplored. While OD has been shown to influence online behaviour in both aggressive and prosocial directions (12), its differential impact on specific CDA subtypes has not been systematically examined. Similarly, although OSP strategic approaches and online platform use frequency have been associated with broader forms of cyberaggression (11, 62), their targeted associations with CDA perpetration remains empirically untested.

To address these gaps, the present study investigates how OD, OSP, and platform use frequency contribute to CDA perpetration across two distinct subtypes: Direct Aggression and Cyber Control and Monitoring. This approach not only clarifies the psychological theories and variables associated with CDA but also informs preventative strategies by identifying modifiable digital behaviours. In addition to these CMC variables, relationship satisfaction is included as a comparative predictor, given its robust and consistent association with CDA (26, 53–55). Lower satisfaction has been linked to heightened emotional dysregulation, including anger and distress (56–58), which may be directed toward romantic partners and manifest as CDA (44, 54, 60, 61). By incorporating relationship satisfaction, the study assesses the relative contribution of interpersonal versus digital communication factors in predicting CDA perpetration. This investigation employs a cross-sectional design using a self-reported online questionnaire to examine the predictive value of these variables. The following hypotheses were tested.

Hypothesis One (H1). “Toxic” OD will positively predict Direct Aggression and Cyber Control and Monitoring perpetration;

Hypothesis Two (H2). “Benign” OD will negatively predict Direct Aggression and Cyber Control and Monitoring perpetration;

Hypothesis Three (H3). “Ideal” OSP will positively predict Direct Aggression and Cyber Control and Monitoring perpetration;

Hypothesis Four (H4). “Consistent” OSP will negatively predict Direct Aggression and Cyber Control and Monitoring perpetration;

Hypothesis Five (H5). Online platform use frequency for general and dating purposes will positively predict Direct Aggression and Cyber Control and Monitoring perpetration; and).

Hypothesis Six (H6). Relationship satisfaction will negatively predict Direct Aggression and Cyber Control and Monitoring perpetration).

## Materials and methods

### Participants

326 participants aged between 18 to 68 years old (71.50% women, 28.50% men, *M age* = 24.47, ± *SD* = 8.52) were recruited between February 2023 and April 2024. Participation was based on opportunity (voluntary participation via online platforms) and snowball sampling. More specifically, participants were recruited online through social media platforms (i.e., Facebook, Instagram, WhatsApp), through a survey exchange website (i.e., SurveySwap), and a study participation scheme at the University of Liverpool for first-year undergraduate Psychology students. To take part, participants needed to be 18 years old or over, have at least one previous or current romantic relationship, be a current or previous social media user, and be fluent in English.

Of the 326 participants, the majority, 48.2% (n = 157), reported being in an exclusive relationship, and 33.1% (n = 108) were single. Regarding general use of social media and dating apps, the majority of participants (72.1%, n = 235) reported using these platforms daily. An additional 11.3% (n = 37) used them a few times a week, 3.4% (n = 11) a few times a month, and 3.7% (n = 12) a few days a year. 9.2% (n = 30) had not used these platforms in over a year. Information about the participant relationships and online platform use for dating purposes is presented in **Table 1**. Ethical approval was granted by the University of Liverpool Ethics Committee (ref: 12043).

**Table 1:** The frequency and percentage of relationship and online platform use variables.

Characteristic	*n*	%
Length of most recent relationship		
Over 2 years	133	40.80
1–2 years	46	14.10
6–12 months	44	13.50
3–6 months	36	11.00
1–3 months	43	13.20
Less than 1 month	18	5.50
Unspecified	6	1.80
Where they met their most recent partner		
In person	162	49.70
Through a friend	55	16.90
Dating application	47	14.40
Social media platform	39	12.00
Other	22	6.70
Frequency of online platform use for dating purposes		
Daily	20	6.10
A few times a week	29	8.90
A few times a month	31	9.50
A few days a year	34	10.40
Had not used these platforms in over a year	114	35.00
Do not use these platforms for dating	98	30.10

### Design

This was a cross-sectional questionnaire study employing a correlational design. Predictor variables included “consistent” and “ideal” Online Self-Presentation (OSP), “toxic” and “benign” Online Disinhibition (OD), frequency of online platform use (general and dating purposes), and relationship satisfaction. Age, gender, and Cyber Dating Abuse (CDA) victimisation were treated as control variables. Outcome variables were Direct Aggression and Cyber Control and Monitoring perpetration.

### Materials

#### Demographics

Three items assessed the participants’ demographics: gender, age, and relationship status.

#### Relationship questionnaire

Six items explored participants’ most recent romantic relationship, including how they met their partner and the length of the relationship. Four of these items assessed distinct facets of relationship satisfaction: happiness (“How do you feel about your current or most recent romantic relationship?”), closeness (“How close do/did you feel to your romantic partner?”), emotional satisfaction (“How emotionally satisfying has your current or most recent romantic relationship been?”), and openness (“How often do/did you feel you could open up to your romantic partner?”). Scores for each facet were summed to produce an overall relationship satisfaction. These items were developed by the authors, drawing inspiration from previous studies’ items on relationship satisfaction (63–65). The aggregated measure demonstrated good internal consistency (α = .88).

#### Online dating and social media use questionnaire

Two items asked participants how frequently they used online platforms (i.e., social media or dating applications) for general uses and for dating purposes.

#### Presentation of online self-scale

The Presentation of Online Self scale (POSS; 31) measures how participants present themselves in online contexts. The “ideal” and “consistent” OSP subscales were used in this study. The “ideal” OSP subscale consists of 9 items (i.e., “I feel more comfortable behaving how I want to online”). The “consistent” OSP subscale includes 4 items (i.e., “I feel my personality online is the real me”). The items are scored on a 5-point Likert scale, with 1 - being strongly disagree to 5 - being strongly agree. Greater scores indicate being higher on these traits. The internal consistency of both the “ideal” and “consistent” OSP subscales was acceptable (66) (“ideal” OSP α = .82; “consistent” OSP α = .62).

#### Online disinhibition questionnaire

The Online Disinhibition Questionnaire (ODQ adapted; 67) includes 11 items and measures “toxic” and “benign” OD. It was originally used to examine cyberbullying (68); therefore, an adapted version of the scale by Zhong et al. (67) was used, whereby the “toxic” OD items focus on online sexual aggression. This makes it more suitable in the context of this study, as online forms of sexual aggression are encompassed by CDA (69). The “toxic” OD subscale includes 4 items (i.e., “It is easy to write sexually harassing messages online because there are no repercussions”). The “benign” OD subscale consists of 7 items (i.e., “The Internet is anonymous, so it is easier for me to express my true feelings or thoughts”). Items were scored on a 4-point Likert scale (1 being “strongly disagree” and 4 being “strongly agree”). The internal consistency of “benign” OD was modest (α = .65), this is below conventional thresholds however, it may be considered acceptable in exploratory research with short scales (66). The “toxic” OD subscale yielded a low value (α = .49), which is below psychometric standards for reliability. This may reflect the adapted nature of the items or the sensitivity of the construct. Cronbach’s alpha is also sensitive to the number of items in a scale, with shorter scales more likely to produce lower values (70, 71).

To further examine the construct validity of the “toxic” OD subscale, a factor analysis was conducted following Taber (72). The sampling adequacy was acceptable (KMO = .57) and Barlett’s test of sphericity indicated sufficient inter-item correlations (X^2^(6)=113.04, *p*<.001). Factors with Eigenvalues >1 were retained (73) and loadings >.32 were considered significant (74). The factor analysis identified two interpretable factors explaining 68.23% of the variance: Factor 1 (justification and action) included the items ‘writing unwanted sexual posts online is not bullying’ (.70) and ‘I don’t mind writing unwelcome sexual comments about others online, because it’s anonymous’ (.62); Factor 2 (general beliefs) included ‘it is easy to write sexually harassing messages online because there are no repercussions’ (.57) and ‘there are no rules online therefore you can do whatever you want’ (.50). Despite the low internal consistency, the factor structure supports the theoretical coherence of the “toxic” OD construct, which encompasses both behavioural tendencies and permissive beliefs shaped by online anonymity and lack of accountability (12). We acknowledge this limitation and interpret findings involving this subscale with caution.

#### Cyber dating abuse questionnaire

The Cyber Dating Abuse Questionnaire (CDAQ; 4) is a 40-item scale, with 20 items measuring CDA perpetration and 20 items examining CDA victimisation. Each subscale is made up of 11 items to measure Direct Aggression (i.e., “I have threatened to physically hurt my partner or former partner with new technologies”) and 9 items to explore Cyber Control and Monitoring (i.e., “I have used new technologies to control where my partner or ex-partner has been and with whom”). The items are scored on a 6-point Likert scale, with 1 being “Never” and 6 being “Usually”. The internal consistency of each subscale was acceptable (Direct Aggression perpetration α = .93; Cyber Control and Monitoring perpetration α = .82; CDA Victimisation α = .96).

### Procedure

The questionnaire was developed and administered using Qualtrics. Upon accessing the study link, participants were presented with an information sheet which included a description of the study, the eligibility criteria and ethical considerations. Participants then completed the demographic items, relationship and online platform use questions, the Presentation of Online Self Scale (POSS; 31), the Online Disinhibition Questionnaire (ODQ adapted; 67), and the Cyber Dating Abuse Questionnaire (CDAQ; 4). Participants were asked to provide their answers to the questionnaire based on their most recent or current romantic relationship. Completion time averaged approximately 20 minutes.

### Statistical analysis

Data was analysed with IBM SPSS Statistics 27. Several variables, such as age, general online platform use frequency, CDA victimisation score, Cyber Control and Monitoring perpetration and Direct Aggression perpetration, exhibited high positive skew (skewness > 1). Spearman rank correlations were used for descriptive analyses, as they are less sensitive to skewed distributions (75). Outliers were retained to avoid inflated Type 1 error rates (76).

Two multiple regressions were conducted to explore the influence of the predictor variables on Direct Aggression and Cyber Control and Monitoring perpetration. Aggregated scale scores were treated as interval-level data (77). Despite some skewness of standardised residuals, regression assumptions were considered sufficiently met given the large sample size (77). No multicollinearity or influential cases were detected (78). To improve residual normality, both outcome variables were log-transformed (Log_10_). Due to heteroscedasticity, Weighted Least Squares (WLS) multiple regression was employed (79). CDA victimisation was recorded as a nominal variable, and age, which was identified as a key source of heteroscedasticity, was used as the weighting variable.

## Results

### Descriptive statistics

Spearman’s rank correlations were initially performed to explore the relationship between all variables. Direct Aggression and Cyber Control and Monitoring perpetration were significantly weakly positively correlated with “ideal” OSP and “benign” and “toxic” OD. Online platform use for dating purposes and relationship satisfaction had a weak negative correlation with Direct Aggression only. **Table 2** provides the correlation results and descriptive statistics.

**Table 2:** Correlations and descriptive statistics of the included variables (Mean and SDs, n = 326).

Variable	*M*	*SD*	1	2	3	4	5	6	7	8	9	10	11	12
1. Cyber Control Perpetration	13.60	5.67	–											
2. Direct Aggression Perpetration	12.78	5.60	.42***	–										
3. Age ^b^	24.47	8.52	-.07	-.15**	–									
4. Gender^a^ (F:M)	233:93	–	-.15	-.01	.17**	–								
5. CDA Victimisation	28.25	14.90	.48***	.40***	-.12	-.08	–							
6. "Ideal" OSP	25.16	6.25	.19***	.22***	-.10	-.02	.16	–						
7. "Consistent" OSP	13.24	2.86	-.14	-.11	.01	-.05	-.01	-.03	–					
8. "Benign" OD	16.64	3.19	.17**	.17**	-.04	.05	.10	.52***	-.12	–				
9. "Toxic" OD	6.10	1.91	.17**	.18**	.03	.16	.05	.17**	-.13	.36***	–			
10. Online Platform Use – General	1.67	1.30	-.06	.07	.16**	.04	-.01	-.05	-.04	-.11	.07	–		
11. Online Platform Use – Dating	4.49	1.52	-.15	-.23***	.09	-.02	-.20***	-.21***	.04	-.19**	.01	.01	–	
12. Relationship Satisfaction	15.10	3.75	.01	-.20***	.02	.02	-.13	-.02	.07	.01	.02	-.09	.31***	–

A Bonferroni correction was applied, *p*  = .004 or below were considered significant. The Mean (*SD*) for Cyber Control and Monitoring and Direct Aggression perpetration were calculated prior to transforming the variables. Correlations took place using the transformed Cyber Control and Monitoring and Direct Aggression perpetration variables. CDA victimisation total scores were used in the above correlations rather than the nominal alternative of this variable. Significant coefficients have been placed in bold.

a Indicates a Serial Bipoint correlation was performed due to the variable being a dichotomous nominal variable.

b *n* = 318.

*p<.05, **p<.01, ***p<.001.

### Direct aggression perpetration

A Weighted Least Squares (WLS) multiple regression was conducted to examine the influence of the predictor variables on Direct Aggression perpetration. The overall model significantly predicted Direct Aggression (R^2^ = .23, F (10, 307) = 9.04, *p* <.001), with it accounting for 23% of the variance. “Ideal” OSP (B = .002, (SE = .001), *p* = .017, 95%CI = 0.000 to 0.004), “toxic” OD (B = .022, (SE = .003), *p* <.001, 95%CI = 0.015 to 0.028), and online platform use frequency for general use (B = .009, (SE = .004), *p* = .030, 95%CI = 0.001 to 0.017) significantly positively predicted Direct Aggression perpetration. “Benign” OD (B=-.005, (SE = .002), *p* = .014, 95%CI = -0.009 to -0.001) was a significant negative predictor of Direct Aggression. “Toxic” OD emerged as the strongest predictor (β = 0.386). Each unit increase in “ideal” OSP and online platform for general use was associated with increases of 0.144 and 0.113 units in Direct Aggression, respectively. Conversely, each unit decrease in “benign” OD corresponded to a 0.157 unit increase in Direct Aggression. “Consistent” OSP, online platform use frequency for dating purposes, and relationship satisfaction did not significantly predict Direct Aggression perpetration (see **Table 3**).

**Table 3:** Individual predictors of direct aggression perpetration.

			95% CI			
Variable	Beta	*SE*	*LL*	*UL*	β	*p*
Control variables						
Gender	-.014	.012	-.037	.010	-.059	.255
Age	-.001	.001	-.002	.000	-.080	.139
CDA victimisation	.045	.012	.022	.068	.200	<.001
Predictor variables						
“Ideal” OSP	.002	.001	.000	.004	.144	.017
“Consistent” OSP	-.003	.002	-.007	.001	-.074	.150
“Benign” OD	-.005	.002	-.009	-.001	-.157	.014
“Toxic” OD	.022	.003	.015	.028	.386	<.001
Online platform use – General use	.009	.004	.001	.017	.113	.030
Online platform use – Dating purposes	-.003	.004	-.011	.005	-.039	.473
Relationship satisfaction	-.002	.001	-.004	.001	-.053	.311

### Cyber control and monitoring perpetration

A Weighted Least Squares (WLS) multiple regression was additionally performed to assess the effect of the predictor variables on Cyber Control and Monitoring perpetration. The overall model significantly predicted Cyber Control and Monitoring (R^2^ = .29, F(10, 307) = 12.22, *p* <.001), with it accounting for 29% of the variance. Significant positive predictors included “ideal” OSP (B = .004, (SE = .001), *p* = .007, 95%CI = 0.001 to 0.006) and “toxic” OD (B = .023, (SE = .004), *p* <.001, 95%CI = 0.015 to 0.031). “Consistent” OSP (B=-.007, (SE = .003), *p* = .010, 95%CI = -0.011 to -0.002) was a negative significant predictor of this form of abuse. Concerning the predictor variables, “toxic” OD was the strongest predictor (β = 0.305). Each unit increase in “ideal” OSP was associated with a 0.156 unit increase in perpetration, while each unit decrease in “consistent” OSP corresponded to a 0.128 unit increase in Cyber Control and Monitoring. “Benign” OD, online platform use for general and dating purposes, and relationship satisfaction were non-significant predictors (see **Table 4**).

**Table 4:** Individual predictors of cyber control and monitoring perpetration.

			95% CI			
Variable	Beta	*SE*	*LL*	*UL*	β	*p*
Control variables						
Gender	-.061	.016	-.093	-.030	-.193	<.001
Age	.000	.001	-.001	.002	.004	.944
CDA victimisation	.107	.015	.077	.137	.351	<.001
Predictor variables						
“Ideal” OSP	.004	.001	.001	.006	.156	.007
“Consistent” OSP	-.007	.003	-.011	-.002	-.128	.010
“Benign” OD	-.004	.003	-.009	.002	-.086	.158
“Toxic” OD	.023	.004	.015	.031	.305	<.001
Online platform use – General use	.003	.005	-.008	.014	.027	.548
Online platform use – Dating purposes	-.006	.005	-.016	.004	-.061	.246
Relationship satisfaction	.002	.002	-.001	.006	.064	.206

## Discussion

This study found that greater “ideal” Online Self-Presentation (OSP) and “toxic” Online Disinhibition (OD) significantly predicted higher levels of Direct Aggression and Cyber Control and Monitoring perpetration, supporting the relevant hypotheses. In contrast, lower “consistent” OSP was associated with increased Cyber Control and Monitoring, while lower “benign” OD and greater general online platform use predicted higher levels of Direct Aggression only. These findings partially supported the hypotheses. Notably, relationship satisfaction was not significantly associated with either form of CDA.

### The role of online disinhibition in CDA

Firstly, “toxic” OD was the strongest predictor of CDA perpetration and demonstrated a medium effect (80). This finding aligns with previous research on “toxic” OD and CDA (29), and other forms of cyber aggression (16, 20, 21). The findings also support evidence that CDA is facilitated by the anonymity, invisibility, and physical distance experienced in online environments (22). Despite Stonard (22) only including an adolescent sample, the current study provides evidence that the online environment also influences CDA perpetration within adults.

OD theory proposes that the online environment causes individuals to act uninhibitedly, which may lead to different behaviour than offline (12, 14, 15). CDA may occur due to online anonymity, as it can prevent individuals from taking responsibility for their online actions (12). Additionally, invisibility can cause emotional distancing during online interactions, especially as the victim’s immediate reactions are hidden (68), leading to increasingly aggressive behaviour (17, 81). The asynchronous nature of online interactions further means individuals can leave immediately after sending hostile messages (12). These mechanisms appear particularly salient in explaining the predictive role of “toxic” OD in both overt and covert CDA forms.

Furthermore, previous research suggests that OD, when treated as a unified construct, is not significantly associated with Cyber Control and Monitoring (82). The current study suggests that when differentiating between “toxic” and “benign” OD, a more distinct pattern emerges in terms of their relationships with Direct Aggression and Cyber Control and Monitoring. However, these findings should be interpreted with caution due to the low internal consistency of the “toxic” OD subscale.

Turning to “benign” OD, this dimension was associated with lower levels of Direct Aggression. Characterised by open and expressive online communication (12), “benign” OD may serve as a protective factor against conflict and abuse (83), potentially explaining its negative association with Direct Aggression perpetration. Furthermore, features such as online anonymity and invisibility can encourage individuals higher in “benign” OD to share their emotions and personal experiences (84, 85) which could further reduce the likelihood of aggressive behaviour. Interestingly, “benign” OD was not associated with Cyber Control and Monitoring. Given that “benign” OD is linked to enhanced communication online (51), it may not align with the covert and indirect nature of Cyber Control and Monitoring behaviours (8, 86) which typically do not rely on overt interpersonal interaction.

Although previous research has reported a positive correlation between “benign” OD and CDA (29), the current findings suggest that this relationship may diminish or shift when controlling for confounding variables.

### Online self-presentation and the dynamics of cyber abuse

The small positive association between “ideal” OSP and CDA perpetration aligns with previous research connecting “ideal” OSP to other forms of online aggression, such as online intimidation (34). Strimbu and O’Connell (11) similarly found a positive correlation between “ideal” OSP and cybertrolling, although this association became nonsignificant in regression analyses. This discrepancy may stem from conceptual differences: CDA typically occurs within romantic relationships, where an “ideal” self may be used to attract or maintain relationships (87, 88), whereas cybertrolling often involves anonymous interactions with strangers (89), potentially favouring more fragmented or deceptive forms of self-presentations.

Moreover, the small negative association between “consistent” OSP and Cyber Control and Monitoring aligns with past research, linking difficulties in presenting a “consistent” self to broader patterns of online aggression (35, 36). The covert nature of Cyber Control and Monitoring perpetration may explain its negative association with lower “consistent” OSP, as this abuse can be performed without others knowing (50). Behaviours can include checking the last time a partner used an online platform or using a partner’s passwords to check messages (4). Individuals who struggle to present a “consistent” self online may be less inclined to engage in overtly abusive behaviours and instead resort to more concealed forms of control. This may also account for the lack of association between lower “consistent” OSP and Direct Aggression, which typically involves visible, confrontational acts that occur publicly or in interpersonal settings (86).

The current study measured participants’ OSP preferences directly, which strengthens the link between OSP preferences and CDA perpetration. The findings also argue against the idea that online aggression is more likely to occur because individuals feel liberated to display their negative traits online (15). Alternatively, it appears that presenting inconsistently online can exacerbate CDA perpetration.

Displaying an “ideal” OSP may enhance the feeling of anonymity or invisibility. Presenting in this manner when online can encourage individuals to act more liberated, particularly when posting negative language, which may enhance the OD effect (33). For example, Hu et al. (33) qualitatively found that a prevalent reason for adults to present inconsistently when online was to achieve disinhibition. This interaction could be explored in future research, given that a link between OSP and CDA perpetration was found in this current study. Additionally, displaying an “ideal” OSP can deplete energy due to behavioural monitoring and modification, which can lower self-regulation (37) and potentially lead to greater CDA perpetration (49, 90–92). Taken together, the literature suggests that “ideal” OSP may amplify OD. Future research could explore the dynamic interplay between OSP styles and OD, particularly in romantic contexts where identity management and emotional regulation are critical.

### Online platform use frequency

Greater use of online platforms for general purposes was linked to increased Direct Aggression, but not Cyber Control and Monitoring. In contrast, increased use of online platforms for dating purposes was not significantly associated with either variation of CDA. As this study defined online platforms to include both social media and dating platforms, these findings partially align with Gunnoo et al. (48) who reported a positive association between dating application use and online sexual violence, a behaviour encompassed by Direct Aggression (4). These findings tentatively suggest that general-purpose use may be a more salient driver of CDA than dating-specific platform use. One explanation is that general platform use may increase the likelihood of encountering a partner’s activity incidentally, which may provoke Direct Aggression. As engagement with these platforms intensifies, so does exposure to ambiguous relational cues (38, 44), potentially triggering impulsive responses, including behaviours of Direct Aggression (49).

Contrastingly, Hertlein and van Dyck (50) found partner surveillance (a form of Cyber Control and Monitoring) was associated with dating application use, a result not replicated in the current study. One possible explanation is that Hertlein and van Dyck (50) examined dating applications in isolation, whereas the present study combined dating applications and social media platform use. Dating applications are often used to find casual sexual partners (52), which can trigger possessive jealousy and monitoring behaviours (93), which are incorporated with Cyber Control and Monitoring forms of CDA (4). On the contrary, social media is primarily used to socialise and maintain relationships (47, 51, 94), potentially diluting the predictive power of dating application use alone.

Additionally, the relationship between frequency of online platform use for dating purposes and CDA may be moderated by other factors, such as the perceived importance of online communication with romantic partners. For example, evidence suggests that online communication with romantic partners can foster feelings of closeness among individuals who consider such interactions meaningful (95). This, in turn, may enhance trust (88) and potentially buffer against CDA. However, this effect appears contingent on the perceived value of online communication; Sullivan (95) found that trust was unaffected among individuals who placed less importance on digital interactions with their partner. These individual differences may help explain the absence of a significant association between dating-related platform use and CDA perpetration in the current study.

It is also important to note that the use of a single item measure to assess participant’s online platform use for both general and dating purposes may have limited the sensitivity of the analysis. Single item measures often lack the predictive validity of multi-item scales and may not adequately capture the complexity of the construct (96). Future research should thus explore online platform use frequency using multiple items, and the current findings should be interpreted with caution.

### Relationship satisfaction

In the current study, relationship satisfaction, measured as a composite of openness, closeness, emotional satisfaction, and happiness, was initially found to be significantly and negatively correlated with Direct Aggression perpetration only. This partially supports past research linking lower relationship satisfaction to increased CDA perpetration (26, 53–55). It also aligns with theoretical perspectives suggesting that diminished relationship satisfaction may intensify negative emotions toward a romantic partner (56, 57), thereby escalating conflict and abusive behaviours (83, 97). Given that Direct Aggression is typically more reactive and emotionally charged than Cyber Control and Monitoring (49), this may explain its stronger association with reduced satisfaction. However, when controlling for other variables in the regression analysis, relationship satisfaction did not emerge as a significant predictor of either form of CDA. These findings suggest that CMC factors, such as OD and OSP, may play a more central role in CDA perpetration than relational satisfaction alone. This final point should be interpreted cautiously, particularly given the low internal consistency observed for the “toxic” OD subscale.

One possible explanation for the lack of association between relationship satisfaction and CDA is the temporal mismatch in measurement. While CDA was assessed over the past 12 months, participants reported their current relationship satisfaction. It is therefore possible that satisfaction ratings did not correspond to the same relational context in which CDA occurred. Previous studies that found stronger associations between relationship satisfaction and CDA perpetration often used measures assessing general or retrospective satisfaction (26, 53–55). Additionally, the use of a composite measure encompassing distinct facets (i.e., emotional satisfaction, closeness, openness, and general happiness) may have introduced variability not present in more unified or validated scales, potentially contributing to the discrepancy.

### Study limitations

The cross-sectional design of the study limits the ability to infer temporal or causal relationships between variables (78, 98). Notably, CDA perpetration was reported for the past year and predictor variables, such as current relationship satisfaction and online behaviours, were measured contemporaneously. This temporal misalignment complicates interpretation, particularly given that online behaviours and relational dynamics may evolve over time (99). Despite this limitation, it is well-established that abuse within romantic relationships can occur with varying frequency and duration (100) and often persists over extended periods (101). However, restricting CDA measurement to a shorter time period may have obscured important patterns of abuse as past CDA perpetration would be masked. Future studies should adopt a longitudinal design to better capture the temporal dynamics of CDA and its predictors (102).

Another limitation concerns the internal consistency of the adapted “toxic” OD subscale (67), which was notably low. This may reflect construct heterogeneity (78), as the items varied in focus, with some targeting the perpetration of online sexual aggression and others addressing impulsivity or general beliefs about online behaviour. Given the social undesirability of admitting to sexually aggressive behaviour (103), participants may have responded conservatively, as suggested by the lower mean scores of these items, thereby reducing internal reliability. This limitation may have attenuated the observed associations between “toxic” OD and CDA perpetration. As noted in prior research, low internal consistency can obscure the true strength of associations between predictors and outcomes (104), often resulting in underestimated effect sizes (105). To increase the internal reliability and validity of this construct in future research, the original validated “toxic” OD subscale produced by Udris (68) should be incorporated, as this has demonstrated a higher internal consistency (21, 106). Additionally, including attention checks can improve data quality, enhance internal consistency, and reduce careless responding (107–109).

Although Weighted Least Squares (WLS) multiple regressions were employed to address heteroscedasticity, residual variance may still have affected model reliability (79). This is particularly relevant given the distributional characteristics of the CDA perpetration variables. Direct Aggression was highly positively skewed, even after transformation, likely due to its lower prevalence and greater social undesirability compared to Cyber Control and Monitoring (103, 110). These distributional issues underscore the importance of replicating the findings to ensure robustness and generalisability. Despite these limitations, the study offers valuable insights into the psychological and technological predictors of CDA and highlights key methodological considerations for future research in this area.

### Future research and implications

Future research should examine how technological psychological factors, such as Online Self-Presentation (OSP) and Online Disinhibition (OD), interact to influence CDA perpetration. This present study found significant positive correlations between “ideal” OSP and “benign” and “toxic” OD, suggesting that “ideal” OSP may amplify feelings of anonymity and invisibility, thereby intensifying the OD effect (33). Given the observed associations between CDA and distinct OSP styles, future studies are well-positioned to investigate these interactions more systematically.

Moreover, the role of “multiple” OSP in CDA perpetration should be further investigated. Although the current study did not find a significant correlation between “consistent” and “ideal” OSP, prior research suggests that “multiple” OSP may encompass elements of both (11). Given that CDA can involve the use of false profiles (4), “multiple” OSP may be particularly relevant to understanding deceptive or manipulative online behaviours.

Individual differences may also moderate the relationship between CMC factors and CDA. For example, paranoid ideation (i.e., distrusting others and possessing unproven beliefs that others intend to harm; 111) has been linked to both in-person Intimate Partner Aggression (IPA; 112) and CDA (113). Paranoia can lead to higher jealousy, a known precursor to CDA (114), and may also influence OSP, as individuals high in paranoid ideation tend to conceal imperfections (115). Moreover, such individuals may perceive the internet as a safer space to express dysregulated behaviours (116), potentially exacerbating CDA. Future research should explore these interactions to better understand how personality traits intersect with online behaviours in the context of abuse.

The findings of this study may have important implications for CDA prevention programmes. Interventions could incorporate education regarding how online environments foster disinhibition, particularly in romantic communication (17, 22, 33). Raising awareness about the ambiguity of online interactions (38, 44) may help individuals interpret digital cues more accurately and reduce reactive aggression. Promoting open and direct communication strategies (117) could mitigate relational conflict (83) and lower the risk of CDA perpetration (118). By integrating psychological, technological, and relational insights, future research and intervention efforts can more effectively address the complex dynamics of CDA in digital contexts.

## Conclusion

Overall, this study found that “toxic” OD and “ideal” OSP were positively associated with both CDA perpetration forms. However, lower “benign” OD and greater general online platform use significantly predicted higher Direct Aggression only, while Cyber Control and Monitoring was negatively associated with “consistent” OSP. These findings may suggest that individuals who are psychologically influenced by the online environment, particularly through anonymity, invisibility and physical distance, are more likely to engage in CDA. “Ideal” OSP may amplify these disinhibiting effects, and frequent platform use may increase exposure to ambiguous partner activity, resulting in Direct Aggression. Relationship satisfaction was not significantly related to CDA, indicating that technology-meditated psychological mechanisms may be more influential than relational factors in predicting abusive behaviours. Future research should explore the interaction between these mechanisms and individual differences, such as personality traits or cognitive biases, to deepen understanding of CDA perpetration. The findings of this study also underscore the importance of raising awareness regarding how online environments shape interpersonal dynamics. Prevention programmes should address the disinhibiting effects of digital communication and promote clearer, more intentional relational exchanges to mitigate the risk of CDA.

## Data Availability

The datasets presented in this article are not readily available because ethical approval was granted based on the restriction that only the principal investigator and researchers will have access to the data. Requests to access the datasets should be directed to Rebecca.Ritchie@liverpool.ac.uk.
